# Degeneration Affects Three-Dimensional Strains in Human Menisci: *In situ* MRI Acquisition Combined With Image Registration

**DOI:** 10.3389/fbioe.2020.582055

**Published:** 2020-09-16

**Authors:** Jonas Schwer, Muhammed Masudur Rahman, Kilian Stumpf, Volker Rasche, Anita Ignatius, Lutz Dürselen, Andreas Martin Seitz

**Affiliations:** ^1^Institute of Orthopaedic Research and Biomechanics, Centre for Trauma Research Ulm, Ulm University Medical Centre, Ulm, Germany; ^2^Department of Mechanical Engineering, University of Connecticut, Storrs, CT, United States; ^3^Experimental Cardiovascular Imaging, Department of Internal Medicine II, University Hospital Ulm, Ulm, Germany

**Keywords:** knee, meniscus, degeneration, MRI, image registration, local strain

## Abstract

Degenerative changes of menisci contribute to the evolution of osteoarthritis in the knee joint, because they alter the load transmission to the adjacent articular cartilage. Identifying alterations in the strain response of meniscal tissue under compression that are associated with progressive degeneration may uncover links between biomechanical function and meniscal degeneration. Therefore, the goal of this study was to investigate how degeneration effects the three-dimensional (3D; axial, circumferential, radial) strain in different anatomical regions of human menisci (anterior and posterior root attachment; anterior and posterior horn; pars intermedia) under simulated compression. Magnetic resonance imaging (MRI) was performed to acquire image sequences of 12 mild and 12 severe degenerated knee joints under unloaded and loaded [25%, 50% and 100% body weight (BW)] conditions using a customized loading device. Medial and lateral menisci as well as their root attachments were manually segmented. Intensity-based rigid and non-rigid image registration were performed to obtain 3D deformation fields under the respective load levels. Finally, the 3D voxels were transformed into hexahedral finite-element models and direction-dependent local strain distributions were determined. The axial compressive strain in menisci and meniscal root attachments significantly increased on average from 3.1% in mild degenerated joints to 7.3% in severe degenerated knees at 100% BW (*p* ≤ 0.021). In severe degenerated knee joints, the menisci displayed a mean circumferential strain of 0.45% (mild: 0.35%) and a mean radial strain of 0.41% (mild: 0.37%) at a load level of 100% BW. No significant changes were observed in the circumferential or radial directions between mild and severe degenerated knee joints for all load levels (*p* > 0.05). In conclusion, high-resolution MRI was successfully combined with image registration to investigate spatial strain distributions of the meniscus and its attachments in response to compression. The results of the current study highlight that the compressive integrity of the meniscus decreases with progressing tissue degeneration, whereas the tensile properties are maintained.

## Introduction

Osteoarthritis (OA) of the knee joint is one of the most widespread diseases and therefore of great clinical and socio-economic importance (Woolf and Pfleger, 2003; Altman, 2010). Although OA is mainly considered to be the result of articular cartilage degeneration, research in recent decades have proved OA to be a disorder of the entire joint (Englund et al., 2009). In particular, the semilunar fibrocartilaginous menisci are of utmost importance for the long-term health of the knee joint, because they contribute decisively to load bearing, load transmission and distribution, joint congruity, lubrication, nutrient distribution and joint stabilization (Fox et al., 2015). The mechanical function of the meniscus depends on the structural and molecular integrity of its highly hydrated extracellular matrix, primarily composed of water (∼60–70%), collagen type I (∼15–25%) and a small amount of proteoglycans (PGs) (1–2%) (Herwig et al., 1984; Fithian et al., 1990; Mow et al., 2005). While the negatively charged, sulfated glycosaminoglycan side chains (GAGs), which are attached to the PG core proteins, osmotically attract water and thus contribute to the compression resistance of the tissue, the collagen fibrils arranged in fiber lamellae define the tissue’s tensile strength (Ghadially et al., 1983; Sanchez-Adams et al., 2011). Given the crucial role of the meniscus for healthy knee function, it is also important to understand its role in OA development. Studies have shown that meniscal pathologies, including meniscal tears and degenerative changes of the extracellular matrix, contribute to OA evolution, because they alter the load transmission and distribution to the adjacent articular cartilage (Bolbos et al., 2009; Englund et al., 2011; Guermazi et al., 2013). Therefore, quantitative measurements to detect biomechanical changes in meniscal tissue response under loaded conditions are not only important for the understanding of meniscal physiology, but could also provide a potential valuable diagnostic tool for early stage OA.

Magnetic resonance imaging (MRI) has been widely used to non-invasively detect disorders of the knee joint by identifying distinct deformation characteristics of soft tissues under controlled mechanical loads. Several studies indicated volume and thickness changes of meniscal tissue when comparing unloaded with loaded conditions (Vedi et al., 1999; Tibesku et al., 2004; Kessler et al., 2006), while other MRI studies addressed meniscal movement during weight-bearing and knee flexion (Thompson et al., 1991; Vedi et al., 1999; Kawahara et al., 2001; Boxheimer et al., 2004; Tibesku et al., 2004; Tienen et al., 2005; Yao et al., 2008). Recently, quantitative MRI parameters, including T1ρ and T2 relaxation times, have been evaluated to study the biochemical composition and structural changes of meniscal tissue due to progressive OA (Son et al., 2013; Subburaj et al., 2015; Calixto et al., 2016; Hornakova et al., 2018). MRI has also been used to evaluate the extrusion of menisci in healthy and degenerated knee joints (Berthiaume et al., 2005; Stehling et al., 2012; Patel et al., 2016). To our knowledge, there is no study investigating continuous strain distributions of menisci with a varying degree of degeneration *in situ* under compressive loading using MRI. Therefore, the most likely inhomogeneous strain response of menisci might provide useful insights on their complex structural properties. Moreover, identifying characteristic structural differences might reveal an association between the biomechanical function and meniscus degeneration and could, therefore, potentially help to detect early meniscus degeneration or locations with a higher risk for structural damage.

The complex microstructure of the meniscus as well as inhomogeneous regional patterns in its composition are responsible for directional and zonal differences in tissue response due to functional loading (Jones et al., 1996; Nakano et al., 1997; Messner and Gao, 1998). While the inner, avascular region features a relatively high PG content and experiences high compressive stresses under mechanical loading (Nakano et al., 1997), the peripheral, vascularized region is mainly composed of circumferentially orientated collagen type I fibers which are mainly exposed to tensile hoop stresses (Jones et al., 1996; Messner and Gao, 1998). Furthermore, the asymmetrical shape between the lateral and medial meniscus and the anterior and posterior horns contribute to regional differences during loading and knee joint flexion. Consequently, the inhomogeneous and anisotropic nature of the meniscus further complicates its degeneration progression in the context of knee joint OA, because the disease can affect any region differently than any other.

Therefore, the objective of this study was to investigate how progressing degeneration effects the three-dimensional (3D: axial, circumferential, radial) strain in different anatomical regions of human menisci and their root attachments under subject-specific bodyweight (BW). In an earlier study (Freutel et al., 2014), high-resolution MRI was successfully combined with intensity-based image registration to obtain the displacement and local strain of porcine medial menisci under axial compressive load using an MRI-compatible *in situ* loading device. For the objective of the current study, this method was adapted to assess 3D strains in the menisci of human cadaveric knee joints. It was hypothesized that the spatial strain in all anatomical regions of the meniscal tissue increases with progressive knee joint degeneration.

## Materials and Methods

### Study Design

**FIGURE 1:**
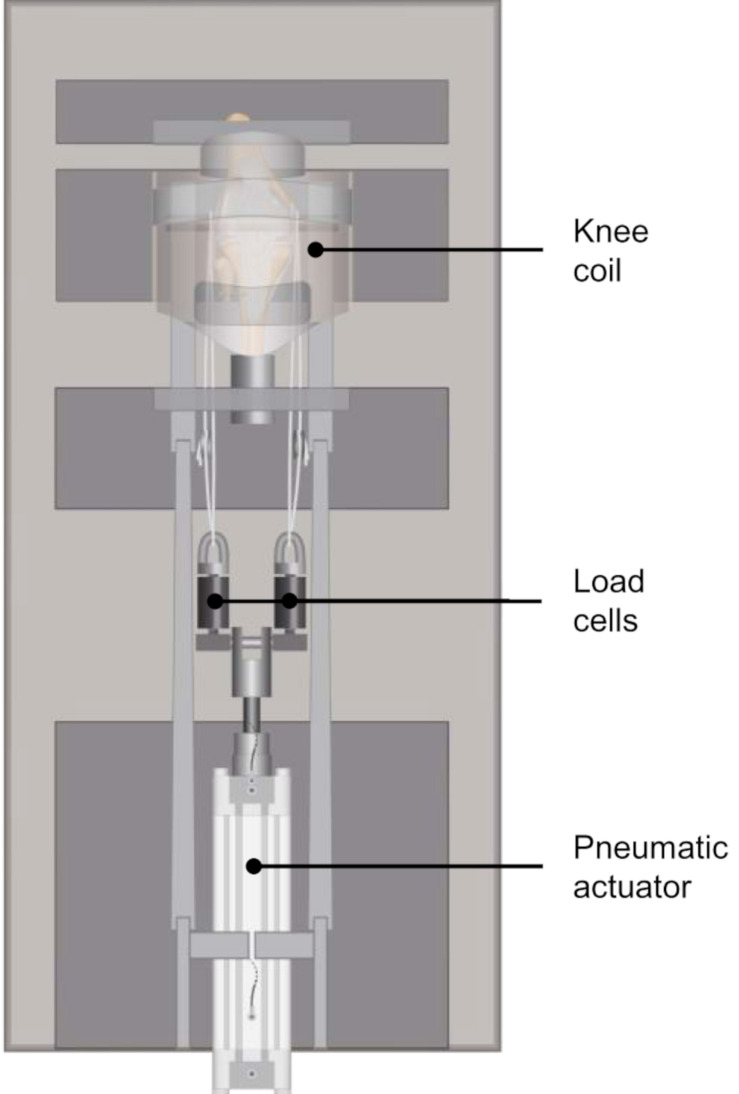
Schematic representation of the modified MRI-compatible loading apparatus introduced by Freutel et al. (2014), equipped with two axial load cells for controlled application of the axial load on the lateral and medial knee compartments.

**FIGURE 2:**
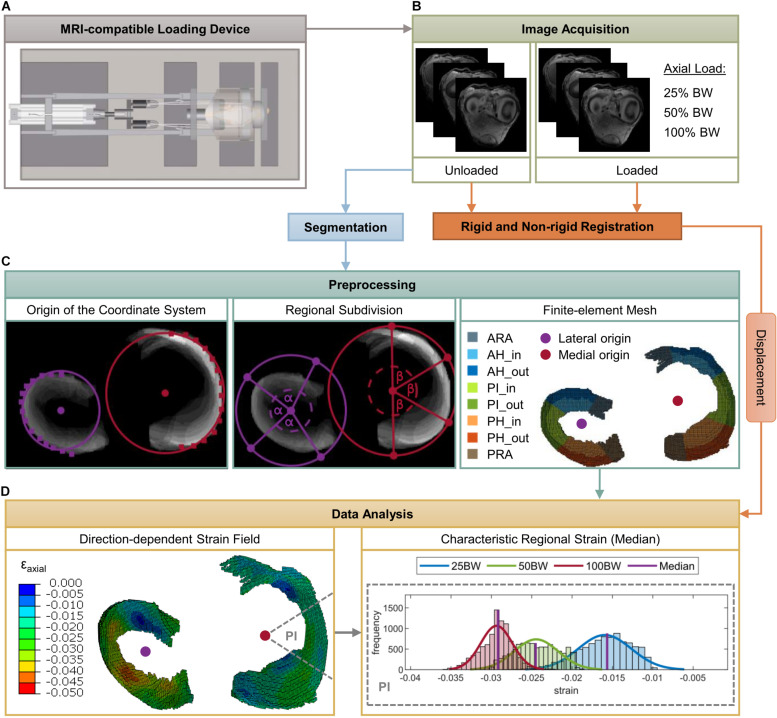
Schematic overview of the methodology of the present study. Mild and severe degenerated knee joints were mounted in an MRI-compatible loading apparatus **(A)** at 5° extension. Subsequently, image sequences were acquired for both unloaded and loaded conditions [25%, 50%, 100% body weight (BW)] **(B)**. Intensity-based rigid and non-rigid registration were performed to obtain the deformation of the menisci under the respective loads. Based on three-dimensional voxel models of the medial and lateral menisci, which were obtained by segmentation of the unloaded image sequence, a hexahedral finite-element (FE) mesh was created **(C)**. Furthermore, lateral and medial origins for the definition of cylindrical coordinate systems were determined and the menisci were subdivided into five anatomic regions (ARA = anterior root attachment; AH = anterior horn; PI = pars intermedia; PH = posterior horn; PRA = posterior root attachment) as well as in an inner and outer circumference **(C)**. Finally, nodal displacements were applied to the FE mesh, strain fields in the axial, circumferential and radial directions were calculated and characteristic regional (median) strain values were determined for the subsequent statistical analyses **(D)**.

Mild and severe degenerated human cadaveric knee joints were mounted in an MRI-compatible loading device that allowed the application of a controlled axial load according to the subject’s individual BW (Figure 1). T1-weighted image sequences were acquired for both unloaded and loaded conditions. Rigid and non-rigid image registration were performed to obtain the deformation of the menisci under the respective loads. Following segmentation of the menisci and meniscal root attachments, the 3D voxel models were converted into a hexahedral finite-element (FE) mesh. For each meniscus, a cylindrical coordinate system was created to calculate axial, circumferential and radial strains. The menisci were subdivided into five anatomical regions [anterior and posterior root attachment (ARA and PRA, respectively); anterior and posterior horn (AH and PH, respectively); pars intermedia (PI)] and divided into an inner and outer zone to investigate possible depth-dependent differences in strain response. Based on characteristic strain values for each region and direction, non-parametric statistical analyses were performed (Figure 2).

### Specimen Preparation

Based on the Kellgren-Lawrence (KL) classification (Kellgren and Lawrence, 1957), 24 human cadaveric knee joints (Science Care, Inc. Phoenix, AZ, United States; IRB approval no. 70/16, Ulm University) were equally divided into a mild (KL: 1–2; age: 47 ± 11 years; BW: 71 ± 12 kg) and severe (KL: 3–4; age: 82 ± 9 years; BW: 85 ± 15 kg) degeneration group (Table 1). All knee joints were dissected and subcutaneous soft tissue was removed, leaving the capsuloligamentous structures intact. The proximal femur and the distal tibia were exposed and transected 16 cm above and below the joint gap and the remaining distal tibia was embedded in polymethyl methacrylate (Technovit 3040, Kulzer GmbH, Wehrheim, Germany). In 0° knee extension, a hole was drilled transversally through the femoral condyles to allow the insertion of a carbon rod (Ø 11 mm) for the later application of axial compressive loads. The hole was drilled in accordance with the subject’s individual knee joint rotation axis to achieve an unconstrained axial loading scenario. All knee joint donors were provided fresh frozen. Following preparation, the specimens were frozen at –20°C and thawed at room temperature prior to testing. During preparation and testing, the knee joints were kept moist in phosphate-buffered saline-drenched gauze.

**TABLE 1:** Summary of demographic specimen data for each group (age, weight, height and body mass index (BMI) reported as mean ± standard deviation) and frequencies of morphological degeneration grades according to Kellgren-Lawrence.

Group	Gender (female/male)	Side (left/right)	Age (yrs)	Weight (kg)	Height (cm)	BMI	Kellgren-Lawrence grade (frequency)			
							1	2	3	4
Mild	6/6	6/6	47 ± 11	71 ± 12	177 ± 9	23 ± 3	1	11	−	−
Severe	0/12	5/7	82 ± 9	85 ± 15	175 ± 8	28 ± 4	−	−	9	3
Both	6/18	11/13	64 ± 20	78 ± 15	176 ± 9	25 ± 4	1	11	9	3

### MRI-Compatible Loading Device

A validated, customized MRI-compatible loading apparatus (Freutel et al., 2014) was modified to allow for the application of subject-specific compressive loads to human knee joints. The loading device can be adjusted to account for different specimen sizes and enables the application of an axial load of up to 1.8 kN. Two axial load cells (KM30z, ME-Meßsysteme GmbH, Hennigsdorf, Germany) were attached to the actuating piston rod of the pneumatic cylinder, which allowed for a controlled application of the axial load on the lateral and medial knee compartments (Figures 1, 2A). The knee joints were mounted in approximately 5° knee flexion in the loading device, which was predefined by the knee coil used for image acquisition. Each load cell was firmly connected by a rope to the carbon rod, which was inserted in the drilled hole of the femoral condyles. For the load application, a calibrated, customized pneumatic actuator without ferromagnetic parts was used. Calibration of the pneumatic system was performed during pretests using a material testing machine (Z010, Zwick, Ulm, Germany). The axial load distribution was adjusted to allow for a physiological load distribution on the lateral and medial compartments of approximately 40% and 60%, respectively, by lengthening or shortening of the respective ropes (Sabzevari et al., 2016). This load balance corresponds to a normal knee joint alignment with a femoral-tibial angle of 5–7° valgus and a joint line of 1–3° varus relative to the mechanical axis (Johnson et al., 1980; Yoshioka et al., 1987; Hsu et al., 1990; Tria, 2002).

### Image Acquisition

Image acquisition was performed using a 3T MRI system (Achieva, Philips Medical Systems, Best, The Netherlands), which was equipped with an 8-channel SENSE knee coil (Philips Medical Systems). All MRI scans were acquired using a T1-weighted 3D turbo spin echo (3D TSE) sequence. The pulse sequence parameters were: echo time = 11.7 ms, repetition time = 750 ms and TSE factor = 5. Spatial resolution was set to 0.4 × 0.4 × 0.6 mm^3^ (sagittal × coronal × axial) with an acquisition matrix of 384 × 384 × 83, pixel bandwidth of 265 Hz and imaging frequency of 127.75 Hz, resulting in a total scanning time of 66 min per load condition of a knee joint. For each knee specimen, a set of four sequential 3D TSE series were acquired at different subject-specific load levels (Figure 2B). First, each knee specimen was scanned in its unloaded state, followed by axial compressive loads equivalent to 25%, 50% and 100% BW of the knee joint donor. Under the loaded conditions, image acquisition was initiated after the specimens were subjected to the respective BW level for 15 min to account for the viscoelastic behavior of the soft tissues in the knee joint (Wang et al., 2015).

### Image Registration

Rigid and non-rigid registrations was performed to obtain the 3D displacement of the menisci under the respective BW levels. In the first step, a mask of the anatomic landmarks of the tibia was created regarding the acquired image data of the unloaded condition using customized MATLAB routines (v. 2018b, The MathWorks, Inc., Natick, MA, United States). Subsequently, all image sequences of the loaded conditions (25%, 50%, 100% BW) were rigidly aligned with the generated mask of the tibia using the open source image registration tool ANTS (Avants et al., 2011). This step was necessary to compensate possible tibial deflections due to adjustments of the setup while changing the BW level. In a second step, all rigidly aligned image sequences were manually cropped to a specified region of interest to reduce the computational effort of the subsequent non-rigid registration. In doing so, the same cropping window with equal pixel dimensions was used for all load cases of a specimen, ensuring that the lateral and medial menisci as well as the meniscal root attachments were captured within the cropping window for all images. On the basis of the cropped image stacks, non-rigid registration was performed to quantify the 3D pixel displacements of the menisci and their attachments between the unloaded (fixed) and loaded (moving) image files using ANTS (Avants et al., 2011). Therefore, a diffeomorphic transformation model was utilized using a cross-correlation similarity metric (Avants et al., 2011) with a window radius of 2 and a gradient decent step size of 0.25. To regularize the deformation field, a gaussian filter with a sigma of 3 was applied. The optimization was performed over four resolution levels with a maximum of 1000 iterations at the coarser level and 100 iterations at full resolution (1000 × 500 × 250 × 100). The termination criterion for the energy minimizer was set to 1e–6. These registration parameters were obtained during pretests based on the earlier study with porcine knee joints (Freutel et al., 2014).

### Preprocessing

First, the medial and lateral meniscus as well as their ARA and PRA were manually segmented (AVIZO, v. 9.4.0, FEI Visualization Sciences Group, Burlington, MA, United States) regarding the unloaded image sequence. On the basis of the resulting 3D voxel models, hexahedral FE models (element size corresponds to the image resolution: 0.4 × 0.4 × 0.6 mm^3^) were generated using a custom MATLAB code (Figure 2C). For each medial and lateral meniscus, an individual local cylindrical coordinate system was defined to subsequently evaluate the local strains in the axial, circumferential and radial directions. The origin of each coordinate system was determined by the center of a circle that resulted from interpolation of manually selected points on the outer circumferential border of the meniscal body (Figure 2C). Because the peripheral region of the meniscus consists mainly of circumferentially oriented collagen bundles, the outer rim was chosen to achieve a good azimuth alignment.

To account for an accurate evaluation of the meniscal strain fields, each meniscus and meniscal attachments were divided into five anatomical regions: ARA, AH, PI, PH, and PRA. First, the meniscal attachments (ARA, PRA) were separated from the meniscal body based on a superposition of the segmented MR image slices by a user-specified separation line starting from the origin of the respective coordinate system and ending at a manually chosen point on the outer circumference. Subsequently, the remaining internal angle between the separated meniscal root attachments was divided by three, resulting in the anatomical regions of the meniscal body (AH, PI, and PH) (Figure 2C). This semi-automatic procedure was applied because the different meniscus dimensions and degeneration states made it difficult to accurately separate the ARA and PRA from the meniscus body using a standard routine. For further region-specific investigations, the meniscal body was divided into an inner and outer zone to account for the different organization of the collagen bundles (Figure 2C). Elements within the inner two-thirds of the meniscal width in the radial direction were defined as the inner zone (AH_in, PI_in, PH_in) and the remaining third as the outer zone (AH_out, PI_out, PH_out) (Beaupré et al., 1986).

### Data Analysis

The 3D pixel displacements obtained from the non-rigid registration were converted to nodal displacements according to the image resolution and subsequently applied to the FE mesh as boundary conditions to obtain the strain field of the menisci and their attachments using FE software (Abaqus/CAE v. 2016, Dassault Systèmes Simulia Corp., Johnston, RI, United States). Based on the strain distributions for each lateral and medial meniscus and each specific load level, the median strain in the axial, radial and circumferential directions was determined for each anatomic region (Figure 2D). These characteristic strain values were considered representative for the respective region and direction.

### Inter- and Intra-Variability

Once the registration parameters have been defined the rigid and non-rigid image registration are deterministic processes. Therefore, only the variability of the user-specific (non-automatic) parts of the presented methodology, i.e., segmentation and regional division of the menisci, can impact the regional strain distributions. To systematically investigate the effect of inter- and intra-observer variability, four knee joints (two of each group) were chosen randomly from the cohort. These specimens were used to undergo two additional segmentations and subsequent regional divisions by two observers on different days within a period of two weeks. The spatial overlap between both, segmentations and divided regions (ARA, AH, PI, PH, and PRA) of the medial and lateral menisci was quantitatively assessed using the Dice similarity coefficient (DSC) (Dice, 1945; Swanson et al., 2010). According to Zijdenbos (Zijdenbos et al., 1994), a DSC above 0.7 indicates ‘excellent agreement’ between two regions.

### Statistical Analyses

A Shapiro-Wilk test resulted in not normally distributed strain data. Therefore, non-parametric statistical analyses were performed using SPSS (v.25.0, SPSS Inc., IBM Company, Armonk, United States). To assess the differences of axial, radial and circumferential strains in all anatomic regions between the mild and severe degenerated groups, Mann-Whitney U testing was performed. Furthermore, Friedman testing was conducted to compare the strains of the different meniscal locations (AH vs. PI vs. PH and AH_in vs. AH_out vs. PI_in vs. PI_out vs. PH_in vs. PH_out) within the mild and severe degenerated groups, respectively. When the Friedman test showed statistical differences, the corresponding data were *post hoc* checked using a paired Wilcoxon test with linear *p*-value Bonferroni correction for multiple comparisons. In addition, a paired Wilcoxon test was performed to assess the difference between the meniscal root attachments (ARA vs. PRA). Kruskal-Wallis testing with subsequent linear *p*-value Bonferroni correction was conducted to check for statistical differences regarding the three different load levels (25%, 50%, 100% BW) in each direction and region. For all statistical analyses *p* ≤ 0.05 was considered significant. A *post hoc* power analysis [G^∗^Power v.3.1 (Faul et al., 2007)] based on the axial compressive strain at a load level of 100% BW revealed a power of 82% between the mild and severe degenerated groups, with an alpha error of 0.05 and one-sided significance.

## Results

### Axial Strain

**FIGURE 3:**
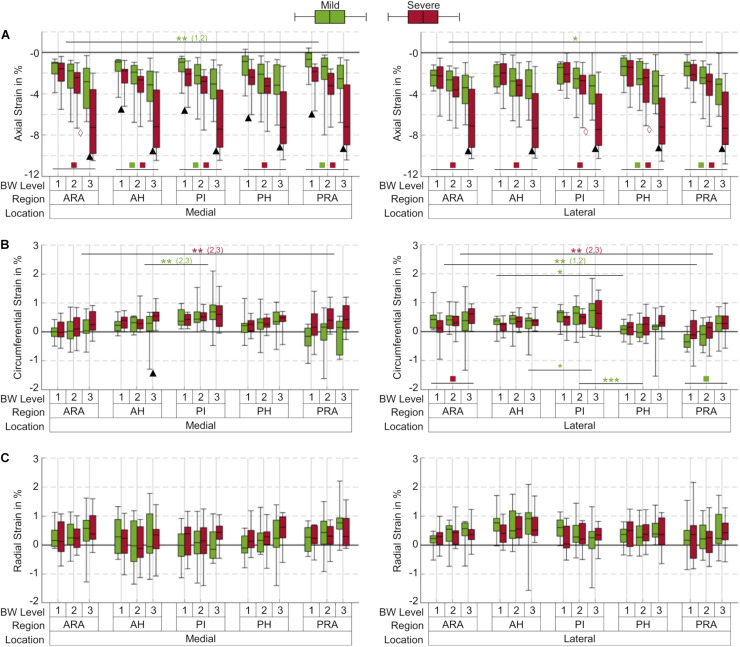
Box plots (maximum, minimum, median, 25–75 percentile values) of axial **(A)**, circumferential **(B)** and radial **(C)** strains at three body weight (BW) levels (1 = 25%; 2 = 50%; 3 = 100% BW) in percent for the mild (green) and severe (red) degenerated group, subdivided into five anatomical regions (ARA = anterior root attachment; AH = anterior horn; PI = pars intermedia; PH = posterior horn; PRA = posterior root attachment). Positive values indicate tension; negative values indicate compression; *n* = 12 for each group and BW level, except for the mild degenerated group at 100% BW (*n* = 10); ▲ indicates *p* ≤ 0.05 between the mild and severe degenerated groups at the respective BW level; *p ≤ 0.05; **(x,y) indicates *p* ≤ 0.05 between the corresponding compartments at BW level x and y regarding the respective group; ***indicates *p* ≤ 0.027 between the corresponding regions for all BW levels within the mild degenerated group; 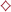 indicates *p* ≤ 0.049 between the BW levels 2 and 3 within the severe degenerated group; 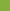 (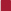) indicates *p* ≤ 0.044 (*p* ≤ 0.002) between the BW levels 1 and 3 within the respective group.

The axial compressive strains in menisci and meniscal root attachments significantly increased by up to 135% in severe degenerated knee joints compared with mild degenerated knees for each anatomical region at 100% BW (*p* ≤ 0.021, Figure 3A). At this load level, mild degenerated menisci exhibited a mean compression of 3.1%, whereas severe degenerated menisci were compressed by a mean of 7.3%. Statistical differences (*p* ≤ 0.039) between mild and severe degenerated knee joints with similar tendency were already apparent in medial menisci at the lowest load level of 25% BW for each region except the ARA. Moreover, the axial compressive strain was significantly increased (*p* ≤ 0.05) for the ARA compared with the PRA in mild degenerated knees at lower load levels (25%, 50% BW medial; 50% BW lateral). Investigating compressive strain under different load levels revealed significantly higher strains (*p* ≤ 0.002) in severe degenerated knee joints for all regions when the load was increased from 25% BW to 100% BW. Regarding the mild degenerated group, similar significant differences (*p* ≤ 0.044) were found for the AH, PI and PRA in medial menisci and for the PH and PRA in lateral menisci. Increasing the load from 50% to 100% BW showed significantly higher strains (*p* ≤ 0.049) in severe degenerated knees in the medial ARA and the lateral PI and PH.

### Circumferential Strain

In general, the circumferential strains in menisci and their attachments were lower compared to the axial compressive strains and depended on the location within the menisci (Figure 3B). The mean circumferential strain at 100% BW in severe degenerated knee joints (0.45%) was slightly higher than in mild degenerated knees (0.35%). Except the medial AH at 100% BW (*p* ≤ 0.05), no significant differences between the mild and severe degenerated groups were found. The highest circumferential tensile strain was observed in the medial PI for both groups (mild: 2.1%; severe: 1.6%) at a load level of 100% BW. Mild degenerated knee joints displayed significantly lower circumferential strains in the AH compared to the PI (50%, 100% BW medial; 100% BW lateral; *p* ≤ 0.05). In addition, the strains in the lateral menisci between the PI and PH significantly decreased for all load levels in the mild degenerated group (*p* ≤ 0.027). Severe degenerated knees displayed a similar tendency, but with no significant change in strain magnitudes. Comparing the circumferential strain between the ARA and PRA showed contrary tendencies for the lateral and medial menisci: While the medial menisci exhibited significantly higher strains in the PRA compared to the ARA in severe degenerated knees (50%, 100% BW; *p* ≤ 0.041), strains in the lateral menisci decreased significantly between the ARA and PRA in these knee joints (50%, 100% BW; *p* ≤ 0.034) as well as in mild degenerated knees (25%, 50% BW; *p* ≤ 0.003). Increasing the load level from 25% to 100% BW resulted in significantly higher circumferential strains solely in the lateral ARA for severe degenerated joints (*p* ≤ 0.024) and in the lateral PRA for mild degenerated knees (*p* ≤ 0.009).

### Radial Strain

**FIGURE 4:**
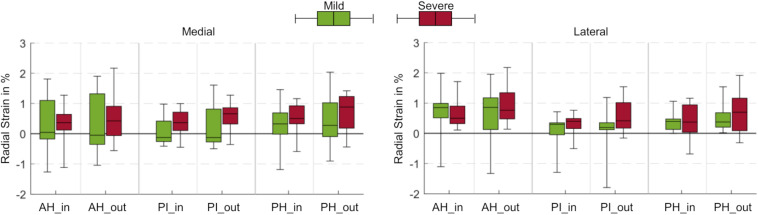
Box plots (maximum, minimum, median, 25–75 percentile values) of radial strains at a load level of 100% body weight in percent for mild (green) and severe (red) degenerated knee joints. Three anatomic regions of the medial and lateral meniscus (AH = anterior horn; PI = pars intermedia; PH = posterior horn) were each subdivided into an inner (_in) and outer (_out) circumference. No statistically significant differences were found (*p* > 0.05).

No statistical differences were observed for the radial strains when comparing data from all load levels and all compartments for both mild and severe degenerated knees (*p* > 0.05, Figure 3C). The mean radial strain at 100% BW in severe degenerated knee joints (0.41%) was slightly higher than in mild degenerated knees (0.37%). Comparison of the regions of the inner and outer circumference at a load level of 100% BW revealed that the median radial strain of the severe degenerated group tended to increase from the inner to the outer circumference, while in the mild degenerated group, comparable high strains occurred in the inner and outer regions (Figure 4).

### Inter- and Intra-Variability

The inter- and intra-observer variation of the manual segmentation and regional division are shown in Table 2. The average inter- and intra-DSC for manual segmentation of both, the medial and lateral meniscus ranged from 0.84–0.88, indicating excellent inter- and intra-observer agreements. Further, all regions except medial ARA and lateral PRA showed an average inter- and intra-DSC above 0.7.

**TABLE 2:** Inter- and intra-observer variation quantified using the Dice Similarity Coefficient (DSC) for both, manual segmentation and subsequent regional division of the medial and lateral meniscus reported as mean ± standard deviation (ARA, anterior root attachment; AH, anterior horn; PI, pars intermedia; PH, posterior horn; PRA, posterior root attachment).

	Medial Meniscus		Lateral Meniscus	
	Intra-DSC	Inter-DSC	Intra-DSC	Inter-DSC
**Segmentation**				
All regions	0,87 ± 0,03	0,84 ± 0,01	0,88 ± 0,03	0,84 ± 0,01
**Regional subdivision**				
ARA	0,68 ± 0,06	0,59 ± 0,13	0,78 ± 0,02	0,71 ± 0,08
AH	0,84 ± 0,05	0,82 ± 0,06	0,85 ± 0,04	0,78 ± 0,07
PI	0,81 ± 0,09	0,77 ± 0,07	0,85 ± 0,05	0,77 ± 0,07
PH	0,82 ± 0,05	0,76 ± 0,09	0,82 ± 0,09	0,70 ± 0,07
PRA	0,78 ± 0,08	0,71 ± 0,11	0,69 ± 0,08	0,52 ± 0,07

## Discussion

The aim of this study was to analyze spatial strain of menisci and meniscal root attachments *in situ* in response to subject-specific axial compressive loading. Subjects with mild or severe degenerated knee joints were investigated focusing on regional differences within the menisci. Therefore, a previously introduced method by Freutel et al. (2014) was adapted for the investigation of human knee joints. The axial compressive strain was significantly higher in severe degenerated knee joints compared to mild degenerated knees for all anatomical regions of the menisci. Degenerative processes might change the phenotype (Rai et al., 2013) of the meniscus including its mechanical properties (Warnecke et al., 2020). Therefore, it was concluded that degeneration might alter the compressive mechanical integrity of the menisci. No significant differences in circumferential or radial strains were found when comparing mild and severe degenerated knee joints, leading to the conclusion that the tensile properties of the meniscal tissue are less affected by progressive degeneration. Therefore, the hypothesis of this study could only be partially confirmed. The findings of the present study rather suggest that progressing degeneration mainly, and perhaps only, affects the compressive properties of meniscal tissue.

Continuous 3D strain fields of both, healthy and degenerated menisci *in situ* under physiological compressive load conditions have to date not been reported. By contrast, several studies have investigated the change of the biomechanical properties of meniscal tissue with varying degeneration (Sandmann et al., 2013; Fischenich et al., 2015). In a comprehensive study, Fischenich et al. (2015) performed indentation relaxation tests as well as tensile tests on human meniscus samples from the anterior and posterior regions and found, that both, the instantaneous and equilibrium moduli significantly decreased with progressing degenerative changes, whereas the tensile modulus was unaffected by degeneration. Furthermore, Sandmann et al. (2013) also performed cyclic indentation tests on meniscus samples and reported lower stiffness in degenerated menisci compared to healthy ones. The present study confirmed these observations, because the higher compressive strain in severe degenerated subjects could be attributed to the lower compressive stiffness and the strains in the circumferential and radial directions were only affected to a minor degree by degeneration. Moreover, Kessler et al. (2015) investigated a continuous strain distribution on meniscal cross-sections of the PH in response to semi confined axial compression using electronic speckle pattern interferometry. They found in the central region of the meniscal cross-section higher strains in degenerative meniscus samples of older subjects compared to healthy meniscus samples, which was also confirmed by our results. A study by Kolaczek et al. (2016) determined 3D (medial-lateral, anterior-posterior and superior-inferior) strain in the PI and PH of the medial menisci (average specimen age: 67 years) by tracking small Teflon markers implanted in the respective central zones using computed tomography imaging at 100% BW and 5° knee flexion. For the PH, they reported an average axial compressive strain of 6.3%, which is consistent with our findings (7.3% for severely degenerated knee joints), but for the PI region they observed an average axial tensile strain of 3.5%, which appears unreasonable due to the axial knee joint loading and therefore contrasts with our data. A further quantitative comparison of the strains with the study by Kolaczek et al. (2016) is not applicable, since different strain directions were evaluated.

Given the changes in compressive strains and the absence of changes in the circumferential and radial directions with progressing degeneration, we think that although the collagen fiber network appears to remain intact and functioning, other tissue components, including GAGs, which are associated with the compressive integrity of meniscal tissue, might be affected (Sanchez-Adams et al., 2011). It is known that GAGs are involved in osmotic swelling, which regulates the pressure of the interstitial fluid and tissue (re-)hydration (Sanchez-Adams et al., 2011). Therefore, under normal loading conditions, the tibiofemoral contact pressure creates an internal stress in the solid phase of the menisci (Mow et al., 2005). When the GAG content is reduced, the fluid flow and retention could be restricted and thus, compressive loads may not be transferred effectively to the collagen fiber network. Because the present data suggest that the collagen fiber network of the degenerated meniscal tissue remains intact, the transfer of compressive load to hoop stress might be compromised. Therefore, future studies should focus on quantifying the GAG content of menisci displaying different states of degeneration and then correlate these data with the compressive and tensile properties determined *in situ*.

Regardless of the degeneration state of the knee joints, much higher strains occurred in the axial compared to the circumferential and radial directions for all load levels. This might be attributable to the anisotropic nature of the meniscus, with a significantly higher stiffness in tension than in compression (Masouros et al., 2008). Increasing the axial load from 25% BW to 50% BW resulted in an average increase of 71% and 48% of compressive strain in mild and severe degenerated knee joints, respectively. However, increasing the axial load level from 25% BW to 100% BW led to a mean increase of 137% and 271%, respectively. Therefore, it can be inferred that the compressive stiffness of the meniscus in mild degenerated knee joints increases non-linearly with increasing load magnitude, whereas it behaves more linearly with progressing degeneration. In addition, the circumferential strains also increased slightly at higher load levels, whereas no distinct pattern was found for the radial strains.

Moreover, the strain response of meniscal tissue not only changed with direction, increased load and degree of degeneration, but also displayed zonal differences. While all regions of the menisci were compressed to almost the same extent in the axial direction, even the ARA and PRA, different strain patterns were observed in the circumferential and radial directions. The highest circumferential strain was found for both medial and lateral menisci in the PI region and decreased slightly in the anterior and posterior directions (Figure 3). In the extension position of the knee joint, the contact area between the femoral condyles, tibial plateau and interposed wedge-shaped menisci is located in the central region of the respective compartment with a slight shift in the anterior direction (Shefelbine et al., 2006). Consequently, most of the compressive load is transferred into hoop strains that appear to load mostly the PI region of the menisci. This load is then transferred via the main collagen fibers to the AH and PH regions, corroborating our findings. In severe degenerated knee joints, we found differences in these loading mechanisms: While the circumferential strain in the root attachments of the medial meniscus significantly increased from anterior to posterior, the circumferential strain of the root attachments in the lateral meniscus significantly decreased from anterior to posterior (Figure 3). Because meniscal degeneration is more common in the PH of the medial meniscus (Zarins et al., 2010), the associated tissue loss might compromise the integrity of the collagen fiber network. Therefore, the forces of impaired collagen fibers could be redirected to intact fibers, resulting in higher strains. However, the significantly higher strain in the ARA of the lateral meniscus might be attributed to the higher compressive load in the anterior region because of the extension position of the knee joints. Furthermore, the radial strains in severe degenerated knee joints tended to increase from the inner to the outer region of both, the medial and lateral meniscus (Figure 4). This observation indicates a higher meniscus extrusion in response to compressive loading due to progressive degeneration, which has already been demonstrated in various studies (Berthiaume et al., 2005; Stehling et al., 2012; Patel et al., 2016).

**FIGURE 5:**
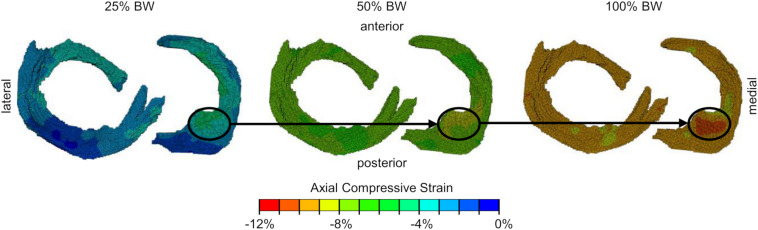
Axial compressive strain fields (from proximal view) of one representative meniscus and its attachments for all three load levels [25%, 50%, 100% body weight (BW)]. Local regions (black circles) with comparably high compressive strains are maintained when applying different load levels.

Several limitations of the present study must be considered when interpreting the results. First, the initial degeneration classification during the selection process of the knee specimens and subsequent grouping was solely based on Kellgren-Lawrence scores without taking demographic data of the donors into consideration. However, these scorings were retrospectively confirmed by a more detailed macroscopic meniscus scoring, according to Pauli et al. (2011) after the knee joints were surgically opened. Unfortunately, the objective selection of specimens without considering gender/age or BMI resulted in a severe degenerated group with exclusively male donors. Furthermore, the average body weight of the severe degenerated group (85 ± 15 kg, Table 1) was higher compared to the mild degenerated group (71 ± 12 kg, Table 1). This resulted in comparably higher axial loads applied to the severe degenerated knee joints. However, the comparison of similar donors with respect to the epidemiological data of both groups revealed significantly higher axial compressive strains in severe degenerated knee joints compared to mild degenerated knees. Therefore, we can conclude that the higher axial compressive strains in severe degenerated knee joints were not caused by the higher average load application. Second, the adjustment of the flexion angle of the knee joints at 5° flexion with slight deviations allowed minor changes in the load transmission between the femoral condyles and the underlying menisci. Furthermore, the distribution of the applied subject-specific load on the medial and lateral knee compartments varied slightly for different specimens and load levels because of the manual adjustment of the rope lengths. Nevertheless, the upgrade of the loading apparatus using two load sensors enabled us to apply an axial load that was more adjusted to the mechanical load axis of human knees (Sabzevari et al., 2016). A qualitative comparison of the strain fields of individual knees with regard to different load levels revealed that local areas in which comparably very high or low strains occur are maintained when the load was increased (Figure 5). This thus supports the good reproducibility of the load application during the experiments. Another limitation of the study relates to its image processing accuracy. Because the non-rigid registration is a deterministic method, the registration accuracy depends on the image quality and resolution. Freutel et al. (2014) superimposed noise on image data and found that the registration accuracy decreased by less than the image resolution, which was more than sufficient to obtain accurate 3D strain fields at the given image resolution of the current *in vitro* study. Moreover, the semi-automatic segmentation of the menisci and the subsequent manual division into different anatomical regions was conducted by a single observer. Both, segmentation and regional subdivision were cross-checked by a second observer. Because of additional information about the meniscal dimensions and appearance after surgically opening of the joints, in three cases, the segmentation and regional subdivision were slightly modified. However, the determined inter- and intra-DSCs (Table 2) demonstrated a high accuracy of the segmentation process and regional division, especially for regions of the meniscal body (AH, PI, and PH). As the rigid and non-rigid image registration are deterministic processes, the high inter- and intra-observer reproducibility of the user-specific processes further indicates a good robustness of the applied methodology. A further limitation is that all the knee joints were only investigated under static knee extension (5° flexion). Changing the knee flexion angle will cause different strain patterns in the menisci and their attachments. Based on the literature investigating meniscal movement during weight-bearing and knee flexion (Thompson et al., 1991; Vedi et al., 1999; Kawahara et al., 2001; Boxheimer et al., 2004; Tienen et al., 2005; Yao et al., 2008), it can be assumed that during flexion the posterior regions would be more affected, than the anterior.

## Conclusion

This *in vitro* study proved that high-resolution MRI in combination with non-rigid image registration is a promising non-invasive approach to study structure-function relationships of the meniscus in human knee joints with different degeneration states. When comparing 3D meniscal strains between mild and severe degenerated knee joints under compression, we found significantly higher axial compressive strains in severe degenerated joints. Circumferential and radial strains were less affected by progressing degeneration. In conclusion, the compressive integrity of the meniscus decreases with progressing tissue degeneration, whereas the tensile properties are maintained. Because the present data suggest that the collagen fiber network of the degenerated meniscal tissue remains intact, one could speculate that the transfer of compressive load to hoop stress might be compromised and should, therefore, be the focus of future studies.

Work is currently ongoing to assess the anisotropic material properties of the individual menisci and meniscal attachments based on the determined 3D strain fields and the applied subject-specific load using inverse FE analyses (Freutel et al., 2015). Additionally, ongoing *in vivo* studies involving healthy volunteers and OA patients will show whether the measurement of 3D local strain in menisci and meniscal attachments can be implemented in a clinical setting. In summary, the non-invasive measurement of 3D strain *in vivo* using a standardized clinical MRI protocol, which necessitates reasonable image acquisition times and sufficient image resolution, combined with the subsequent determination of anisotropic material parameters, might provide a non-invasive diagnostic tool to identify and treat early OA with the final goal to retard or even prevent OA progression.

## Data Availability Statement

The raw data supporting the conclusions of this article will be made available by the authors, without undue reservation.

## Ethics Statement

The studies involving human participants were reviewed and approved by IRB University of Ulm, Germany (no. 70/16). Written informed consent for participation was not required for this study in accordance with the national legislation and the institutional requirements.

## Author Contributions

AS developed and improved the MRI loading device, made substantial contributions to research design, data interpretation, mentoring, and final editing of the manuscript. MR and AS conducted the experiments. KS and VR were responsible for image acquisition during the experiments. JS performed the data analysis and contributed to research design, data interpretation, and drafted the manuscript including all figures. MR contributed to research design and data analysis. LD contributed by conceptualizing, mentoring, fund acquisition, and administrative support. All authors participated in the revision process of the article and gave final approval of the submitted version.

## Conflict of Interest

The authors declare that the research was conducted in the absence of any commercial or financial relationships that could be construed as a potential conflict of interest.
